# Supplementary figures and images for: USP22 as a key regulator of glycolysis pathway in osteosarcoma: insights from bioinformatics and experimental approaches
Source: PeerJ. 2024 May 20;12:e17397. doi: 10.7717/peerj.17397 (PMC11114114; doi:10.7717/peerj.17397)

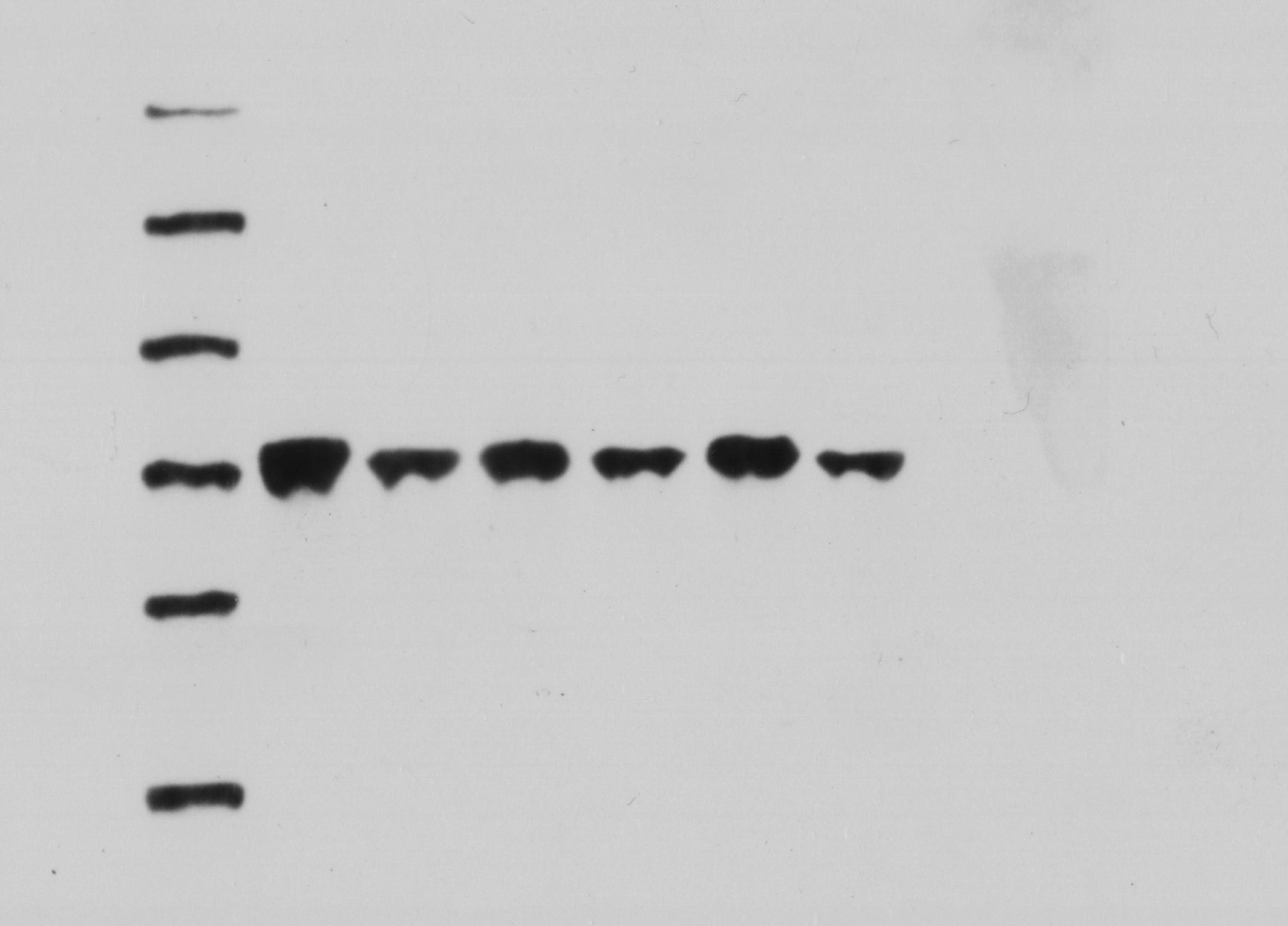

Supplement: Supplemental Information 11 [file peerj-12-17397-s011.jpg]

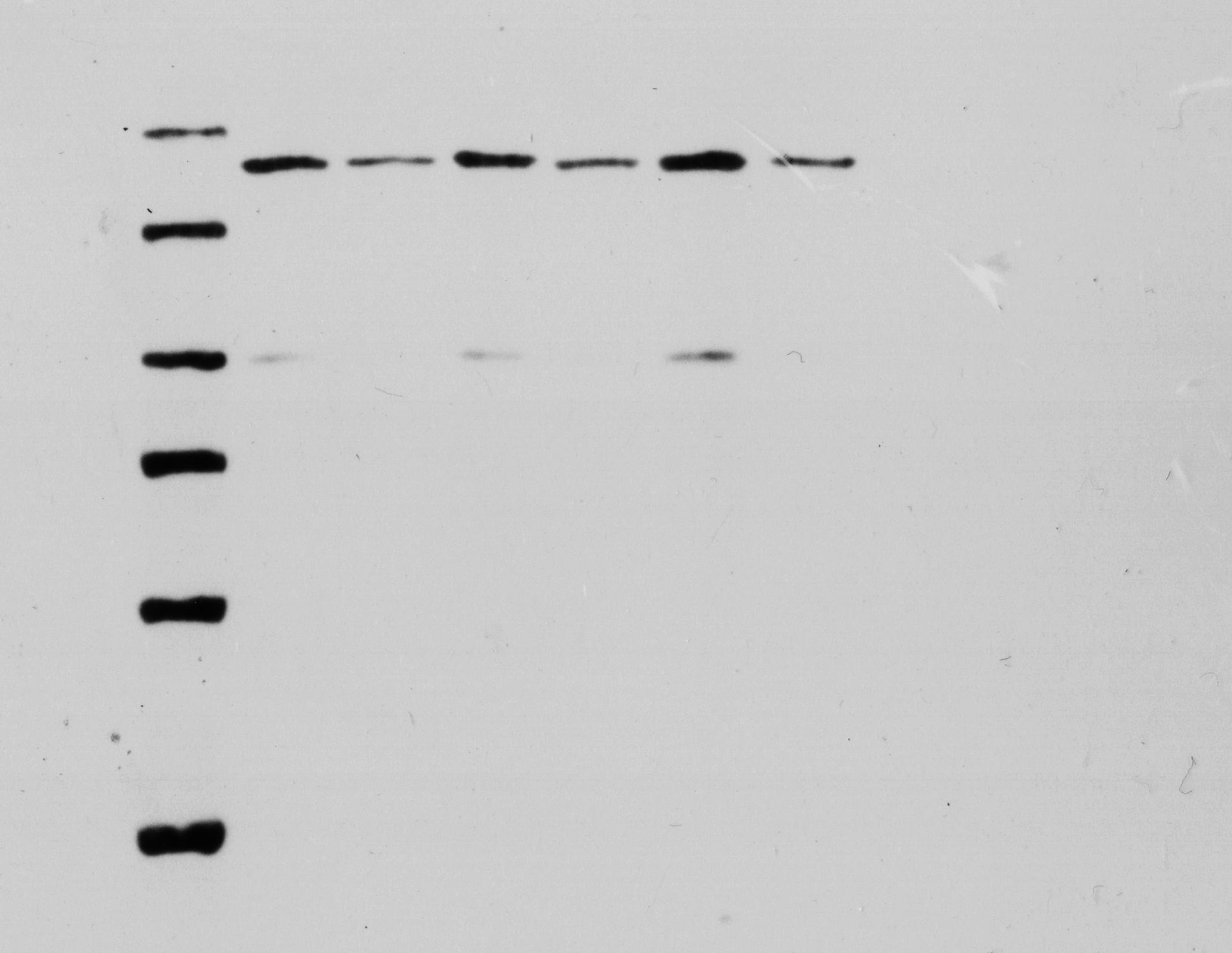

Supplement: Supplemental Information 12 [file peerj-12-17397-s012.jpg]

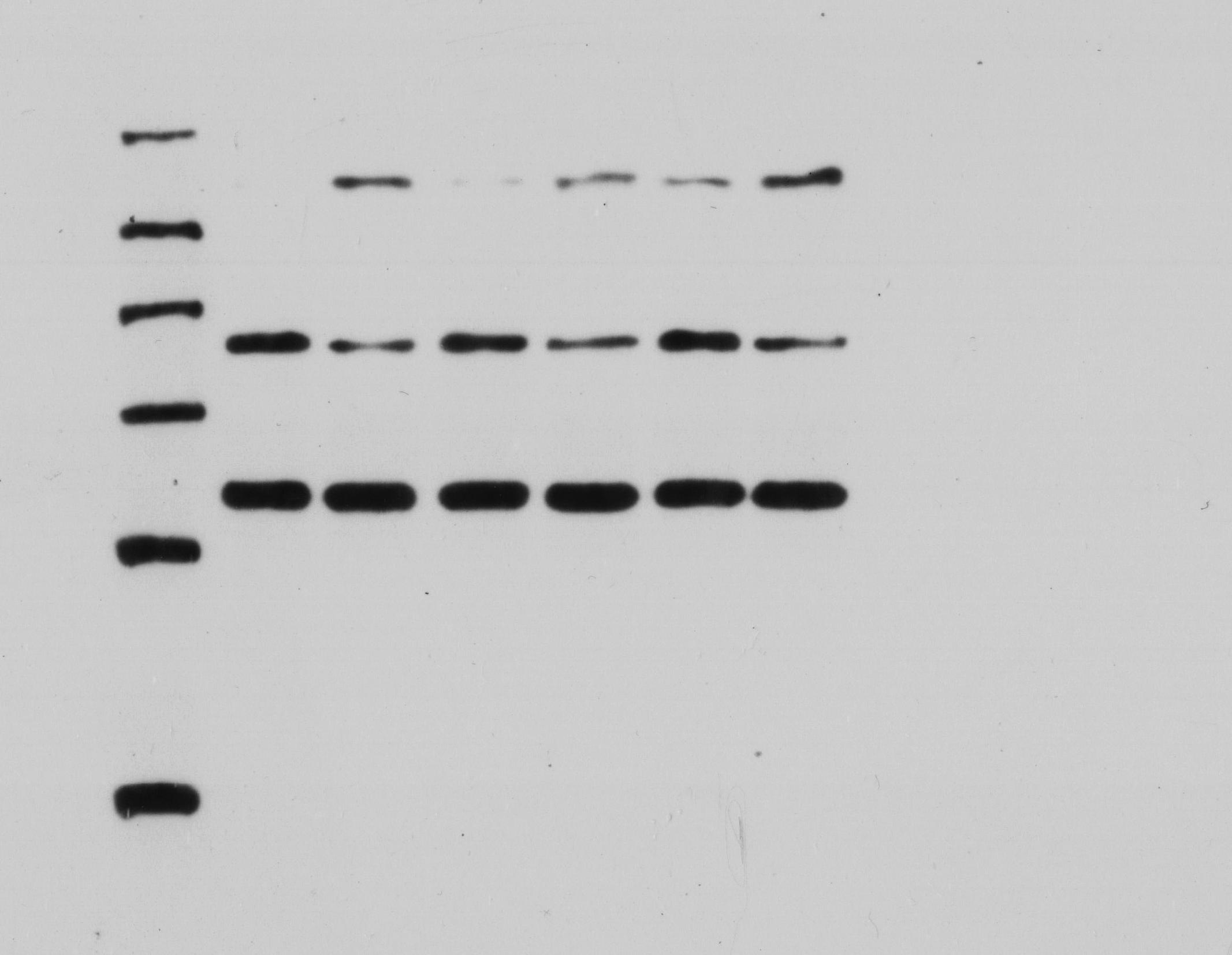

Supplement: Supplemental Information 13 [file peerj-12-17397-s013.jpg]

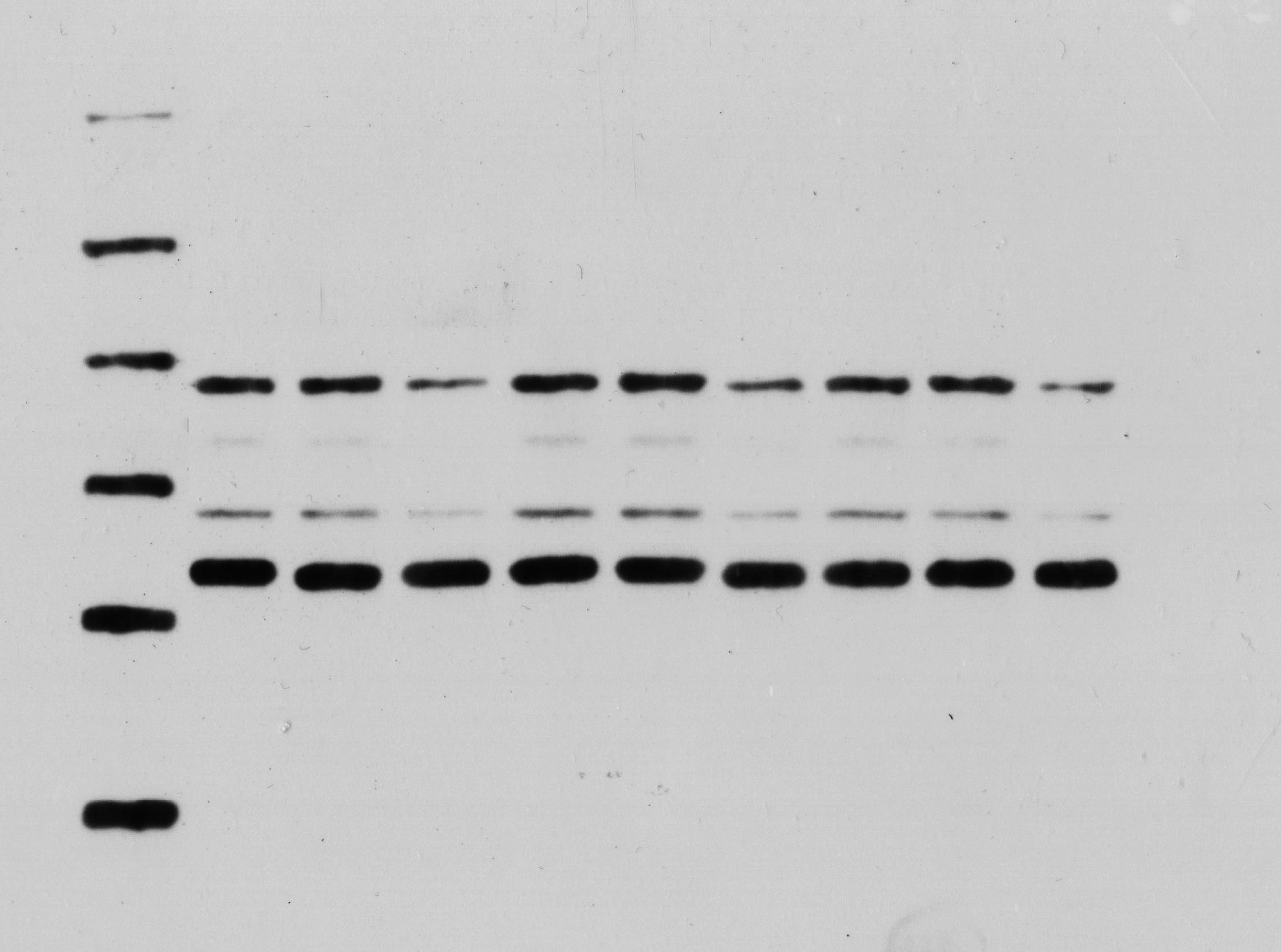

Supplement: Supplemental Information 14 [file peerj-12-17397-s014.jpg]

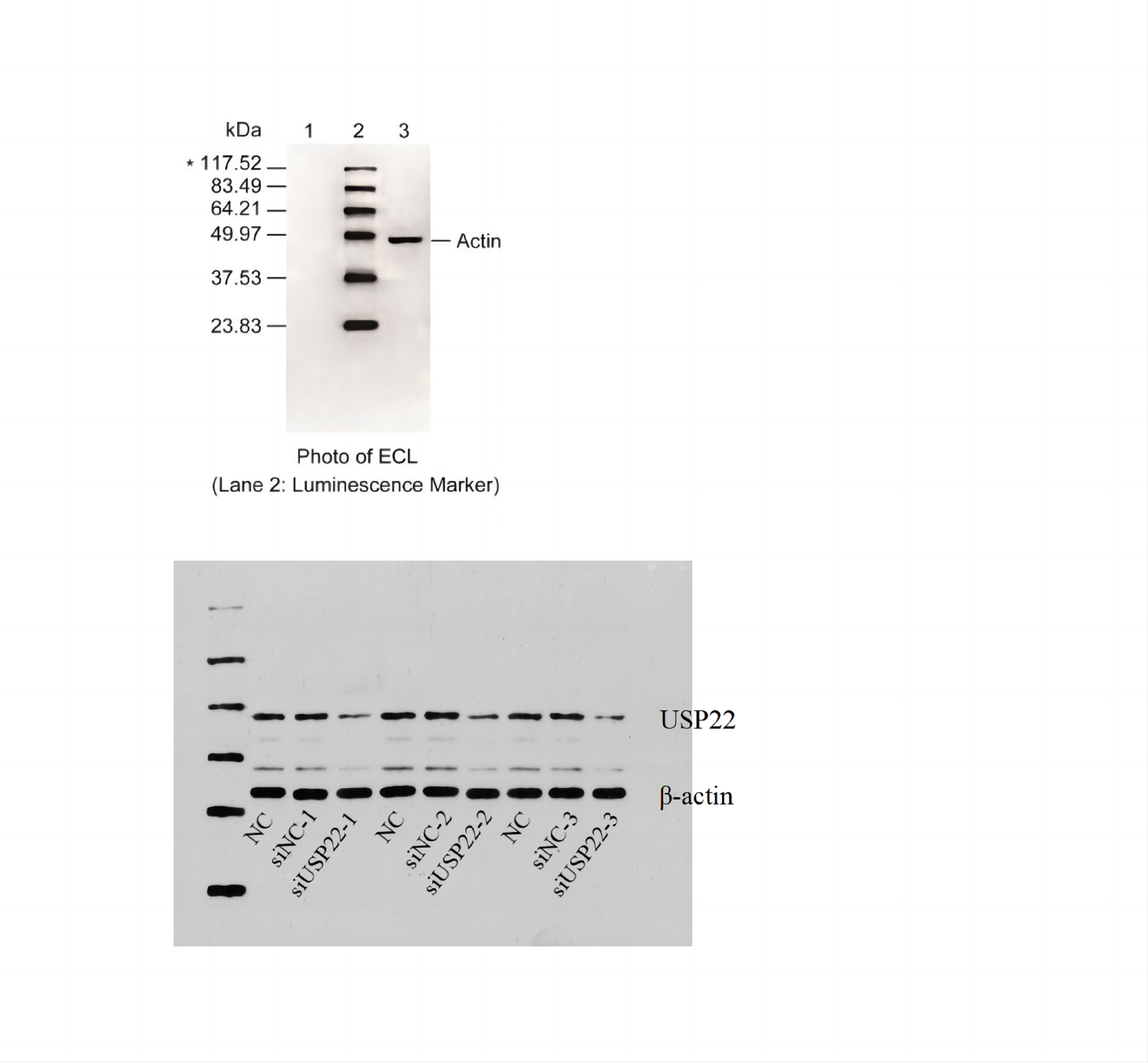

Supplement: Supplemental Information 16 [file peerj-12-17397-s016.png]

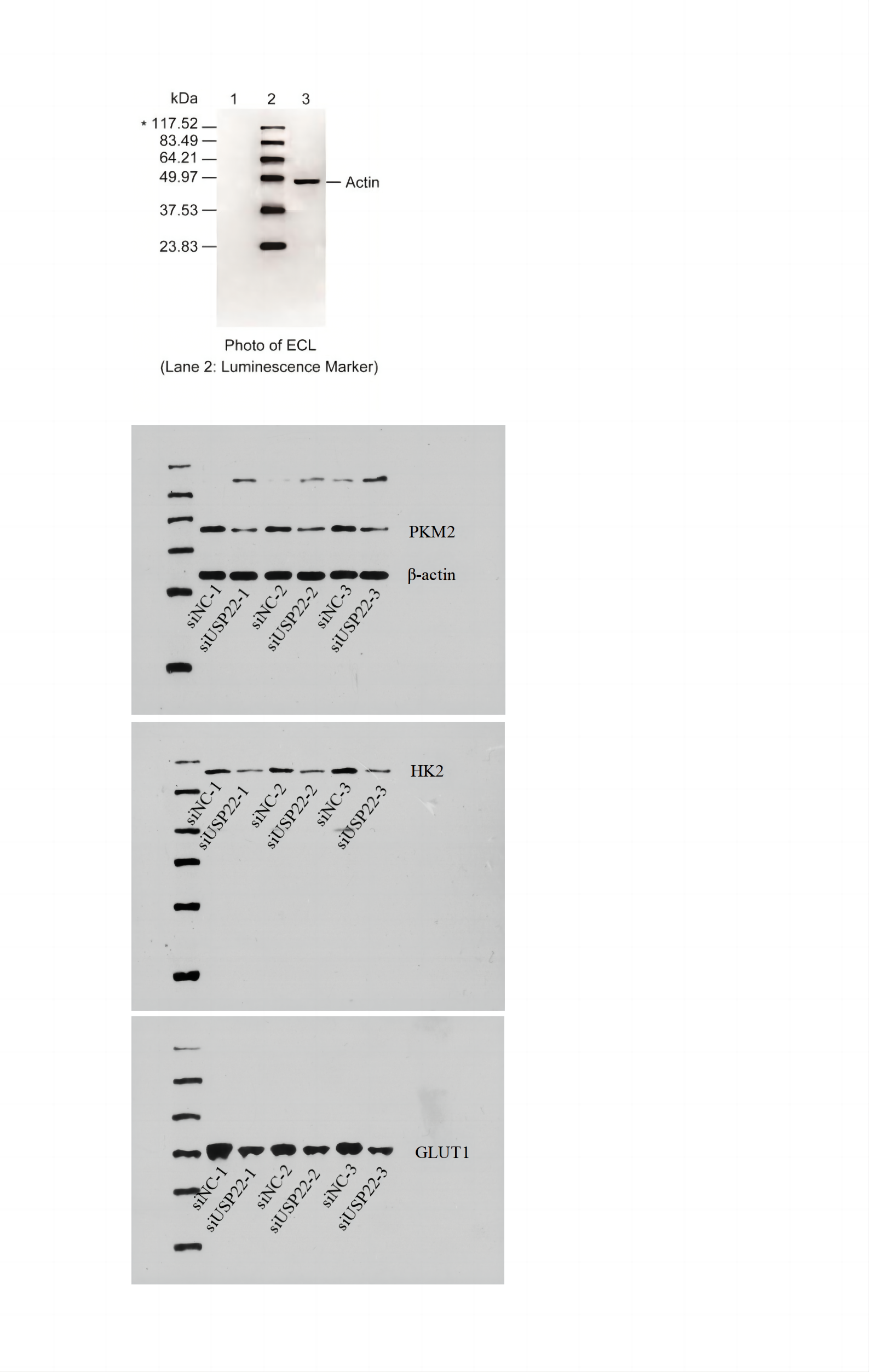

Supplement: Supplemental Information 17 [file peerj-12-17397-s017.png]

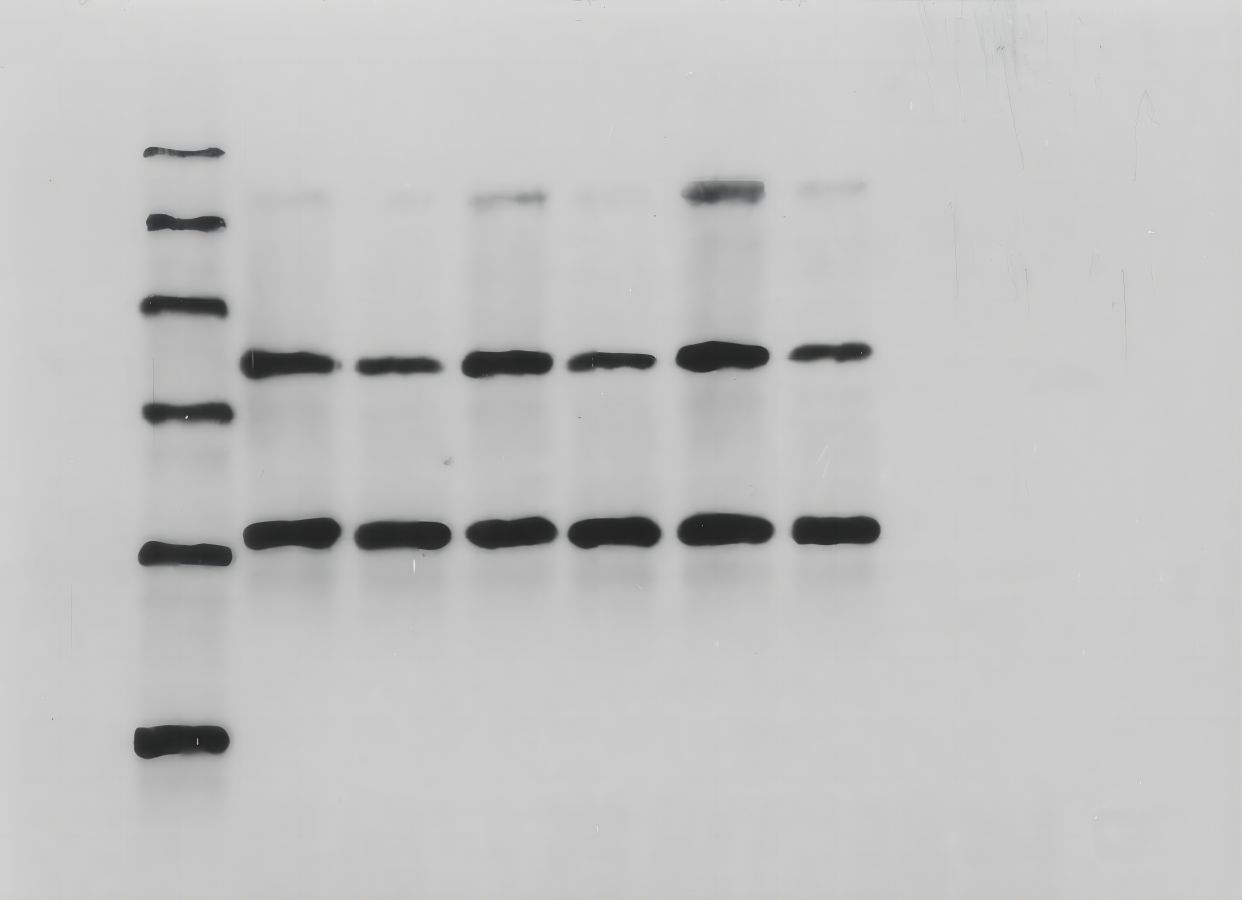

Supplement: Supplemental Information 19 [file peerj-12-17397-s019.jpg]
