# Supplementary material for: USP22 as a key regulator of glycolysis pathway in osteosarcoma: insights from bioinformatics and experimental approaches
Source: PeerJ. 2024 May 20;12:e17397. doi: 10.7717/peerj.17397 (PMC11114114; doi:10.7717/peerj.17397)
Supplement: Supplemental Information 22 — Instrument parameters, gating parameters, and MFI histograms for FACS [file peerj-12-17397-s022.pdf]

Institution:
Protocol: siNC-1.PRO
Listmode Replay: Runtime Protocol
Analysis Date: 20-Feb-2024, 13:18:39
Settings File: hedaliushi230320.PRO, 27-Mar-2023, 16:32:46
Listmode File: siNC-1.LMD

Run Date: 27-Mar-23, 16:33:15
Sample ID: 00012031
User ID: liting
Acquisition Time/Events: 2.7s / 6000 (PROTOCOL)

Instrument SN: RAS11006 Software Version: CXP

(F1)[A] siNC-1.LMD : FS Lin/SS Lin - ADC

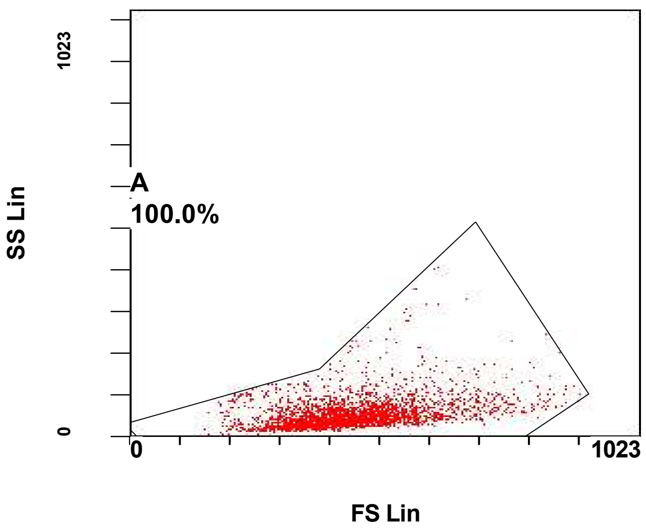

(F1)[A] siNC-1.LMD : FL1 Log/FL3 Log - ADC

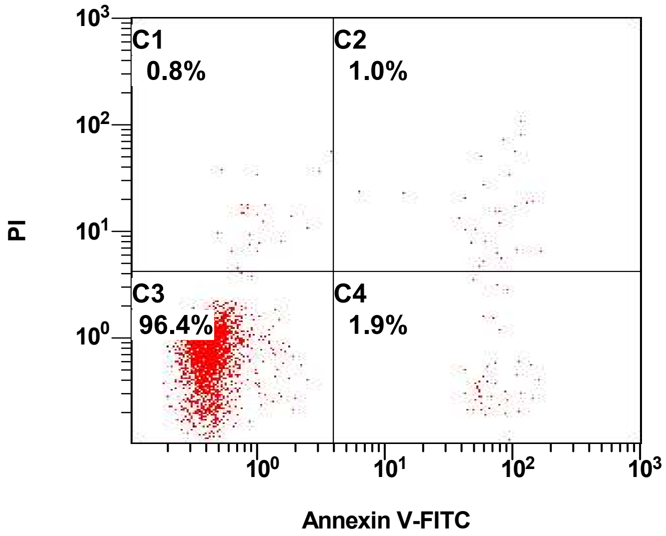

Statistical Analysis

PROGRAM INFORMATION

File:- siNC-1.LMD					
Gate:- A [A]					
Compensation:- Advanced					
Filename:- siNC-1.LMD					
Mean Calculation Method:- LOG-LOG					
Region	Number	%Total	%Gated	X-Mean	Y-Mean
ALL	5534	92.23	100.00	2.42	1.19
ALL	5534	92.23	100.00	455	59.4
A	5534	92.23	100.00	455	59.4
C1	43	0.72	0.78	1.39	16.8
C2	56	0.93	1.01	67.7	24.2
C3	5333	88.88	96.37	0.432	0.838
C4	103	1.72	1.86	70.5	0.691
