# Supplementary material for: USP22 as a key regulator of glycolysis pathway in osteosarcoma: insights from bioinformatics and experimental approaches
Source: PeerJ. 2024 May 20;12:e17397. doi: 10.7717/peerj.17397 (PMC11114114; doi:10.7717/peerj.17397)
Supplement: Supplemental Information 23 — Instrument parameters, gating parameters, and MFI histograms for FACS [file peerj-12-17397-s023.pdf]

Institution:
Protocol: siNC-2.PRO
Listmode Replay: Runtime Protocol
Analysis Date: 20-Feb-2024, 13:18:23
Settings File: hedaliushi230320.PRO, 27-Mar-2023, 16:38:41
Listmode File: siNC-2.LMD

Run Date: 27-Mar-23, 16:39:10
Sample ID: 00012031
User ID: liting
Acquisition Time/Events: 5.1s / 6000 (PROTOCOL)

Instrument SN: RAS11006 Software Version: CXP

(F1)[A] siNC-2.LMD : FS Lin/SS Lin - ADC

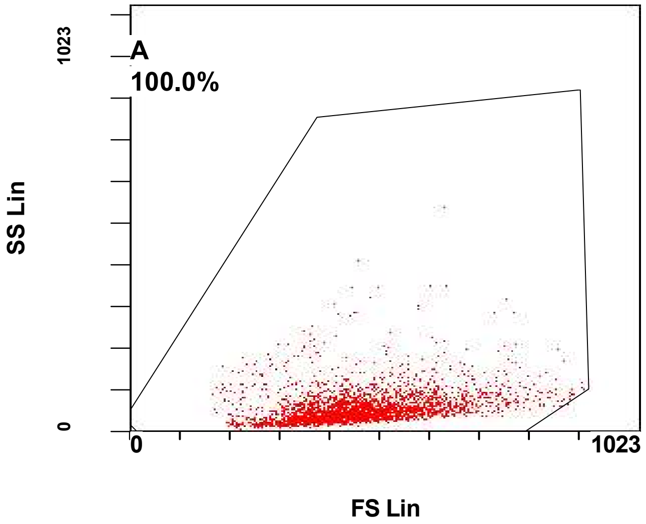

(F1)[A] siNC-2.LMD : FL1 Log/FL3 Log - ADC

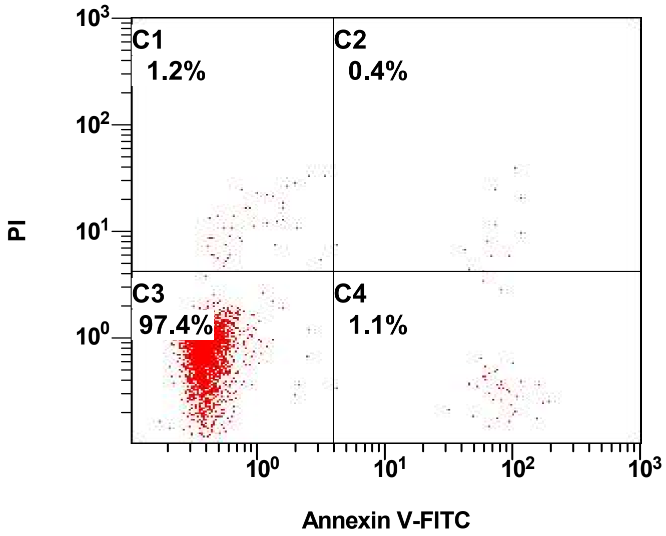

Statistical Analysis

PROGRAM INFORMATION

File:- siNC-2.LMD					
Gate:- A [A]					
Compensation:- Advanced					
Filename:- siNC-2.LMD					
Mean Calculation Method:- LOG-LOG					
Region	Number	%Total	%Gated	X-Mean	Y-Mean
ALL	5939	98.98	100.00	1.49	1.03
ALL	5939	98.98	100.00	459	59.9
A	5939	98.98	100.00	459	59.9
C1	70	1.17	1.18	0.955	12.3
C2	21	0.35	0.35	70.4	14.8
C3	5785	96.42	97.41	0.405	0.854
C4	63	1.05	1.06	78.4	0.467
