# Supplementary material for: USP22 as a key regulator of glycolysis pathway in osteosarcoma: insights from bioinformatics and experimental approaches
Source: PeerJ. 2024 May 20;12:e17397. doi: 10.7717/peerj.17397 (PMC11114114; doi:10.7717/peerj.17397)
Supplement: Supplemental Information 24 — Instrument parameters, gating parameters, and MFI histograms for FACS [file peerj-12-17397-s024.pdf]

Institution:
Protocol: siNC-3.PRO
Listmode Replay: New Protocol
Analysis Date: 20-Feb-2024, 13:41:18
Settings File: hedaliushi230320.PRO, 27-Mar-2023, 16:37:29
Listmode File: siNC-3.LMD

Run Date: 27-Mar-23, 16:38:36
Sample ID: 00012031
User ID: liting
Acquisition Time/Events: 5.2s / 6000 (PROTOCOL)

Instrument SN: RAS11006 Software Version: CXP

(F1)[A] siNC-3.LMD : FS Lin/SS Lin - ADC

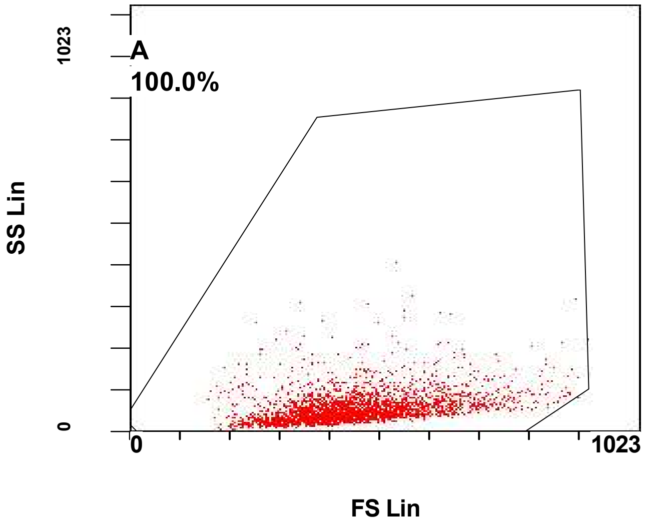

(F1)[A] siNC-3.LMD : FL1 Log/FL3 Log - ADC

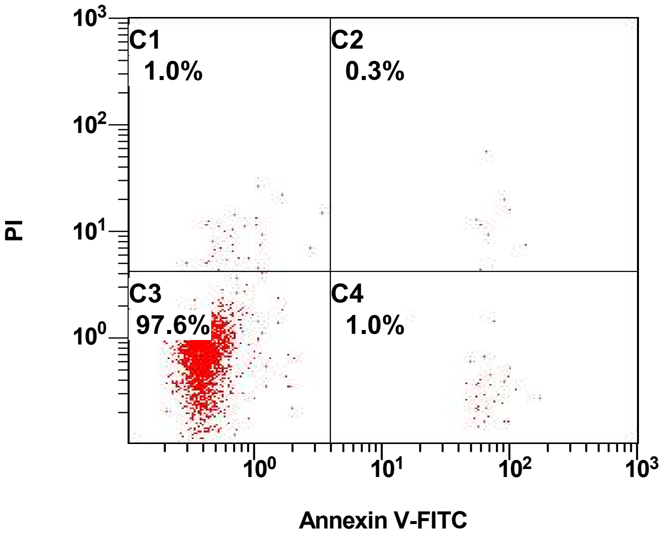

Statistical Analysis

PROGRAM INFORMATION

File:- siNC-3.LMD					
Gate:- A [A]					
Compensation:- Advanced					
Filename:- siNC-3.LMD					
Mean Calculation Method:- LOG-LOG					
Region	Number	%Total	%Gated	X-Mean	Y-Mean
ALL	5950	99.17	100.00	1.41	1
ALL	5950	99.17	100.00	450	60
A	5950	99.17	100.00	450	60
C1	59	0.98	0.99	0.983	10
C2	20	0.33	0.34	68.8	21
C3	5809	96.82	97.63	0.404	0.848
C4	62	1.03	1.04	74.3	0.567
