# Supplementary material for: USP22 as a key regulator of glycolysis pathway in osteosarcoma: insights from bioinformatics and experimental approaches
Source: PeerJ. 2024 May 20;12:e17397. doi: 10.7717/peerj.17397 (PMC11114114; doi:10.7717/peerj.17397)
Supplement: Supplemental Information 25 — Instrument parameters, gating parameters, and MFI histograms for FACS [file peerj-12-17397-s025.pdf]

Institution:
Protocol: siUSP22-1.PRO
Listmode Replay: Runtime Protocol
Analysis Date: 20-Feb-2024, 13:17:54
Settings File: hedaliushi230320.PRO, 27-Mar-2023, 16:26:00
Listmode File: siUSP22-1.LMD

Run Date: 27-Mar-23, 16:26:28
Sample ID: 00011999
User ID: liting
Acquisition Time/Events: 7.6s / 6000 (PROTOCOL)

Instrument SN: RAS11006 Software Version: CXP

(F1)[A] siUSP22-1.LMD : FS Lin/SS Lin - ADC

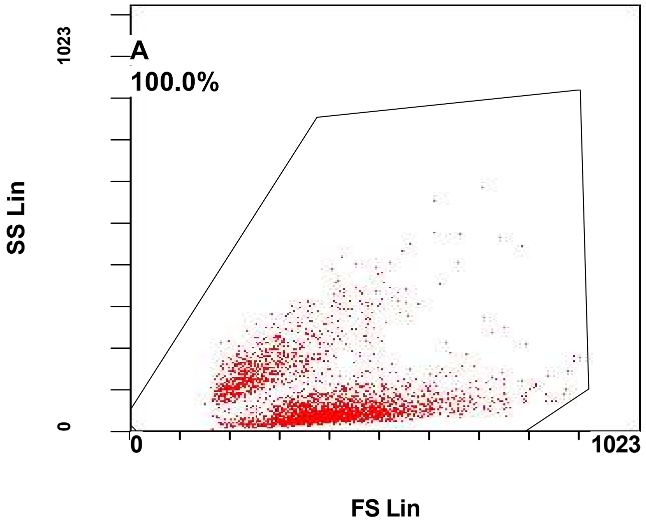

(F1)[A] siUSP22-1.LMD : FL1 Log/FL3 Log - ADC

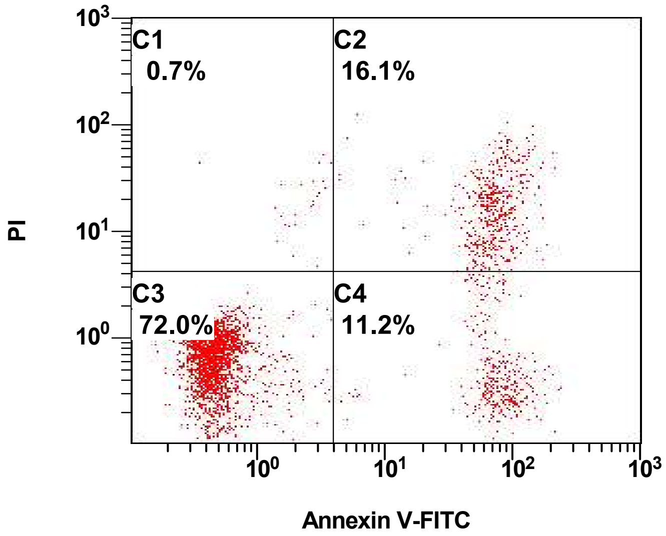

Statistical Analysis

PROGRAM INFORMATION

File:- siUSP22-1.LMD					
Gate:- A [A]					
Compensation:- Advanced					
Filename:- siUSP22-1.LMD					
Mean Calculation Method:- LOG-LOG					
Region	Number	%Total	%Gated	X-Mean	Y-Mean
ALL	5974	99.57	100.00	21.5	4.38
ALL	5974	99.57	100.00	383	85.7
A	5974	99.57	100.00	383	85.7
C1	42	0.70	0.70	2.31	21.7
C2	959	15.98	16.05	74.7	22.4
C3	4302	71.70	72.01	0.498	0.763
C4	672	11.20	11.25	81.2	0.812
