# Supplementary material for: USP22 as a key regulator of glycolysis pathway in osteosarcoma: insights from bioinformatics and experimental approaches
Source: PeerJ. 2024 May 20;12:e17397. doi: 10.7717/peerj.17397 (PMC11114114; doi:10.7717/peerj.17397)
Supplement: Supplemental Information 26 — Instrument parameters, gating parameters, and MFI histograms for FACS [file peerj-12-17397-s026.pdf]

Institution:
Protocol: siUSP22-2.PRO
Listmode Replay: Runtime Protocol
Analysis Date: 20-Feb-2024, 13:17:39
Settings File: hedaliushi230320.PRO, 27-Mar-2023, 16:44:00
Listmode File: siUSP22-2.LMD

Run Date: 27-Mar-23, 16:44:57
Sample ID: 00012031
User ID: liting
Acquisition Time/Events: 2.9s / 6000 (PROTOCOL)

Instrument SN: RAS11006 Software Version: CXP

(F1)[A] siUSP22-2.LMD : FS Lin/SS Lin - ADC

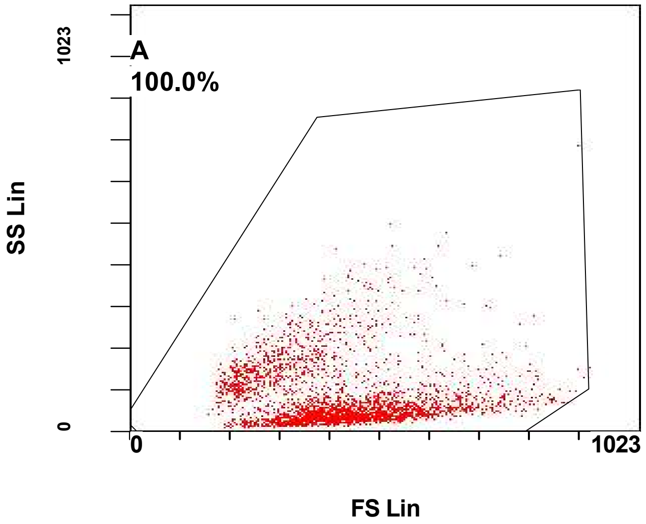

(F1)[A] siUSP22-2.LMD : FL1 Log/FL3 Log - ADC

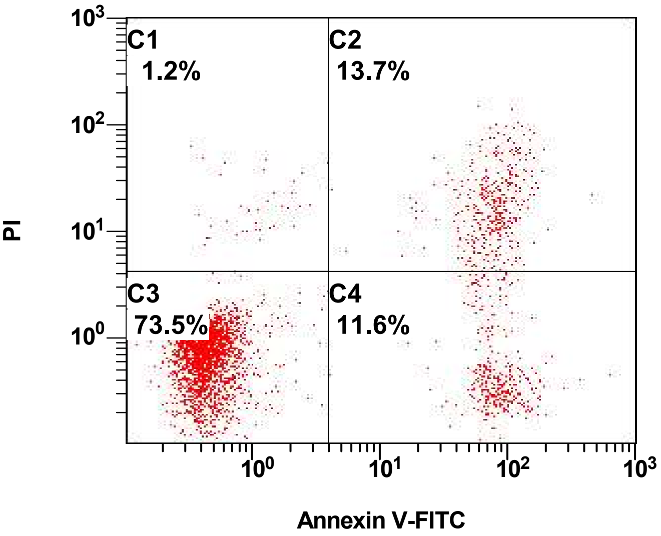

Statistical Analysis

PROGRAM INFORMATION

File:- siUSP22-2.LMD					
Gate:- A [A]					
Compensation:- Advanced					
Filename:- siUSP22.LMD					
Mean Calculation Method:- LOG-LOG					
Region	Number	%Total	%Gated	X-Mean	Y-Mean
ALL	5950	99.17	100.00	21.9	4.35
ALL	5950	99.17	100.00	422	86.9
A	5950	99.17	100.00	422	86.9
C1	70	1.17	1.18	1.23	19.3
C2	815	13.58	13.70	82.7	25.2
C3	4375	72.92	73.53	0.469	0.794
C4	692	11.53	11.63	87.9	0.77
