# Supplementary material for: USP22 as a key regulator of glycolysis pathway in osteosarcoma: insights from bioinformatics and experimental approaches
Source: PeerJ. 2024 May 20;12:e17397. doi: 10.7717/peerj.17397 (PMC11114114; doi:10.7717/peerj.17397)
Supplement: Supplemental Information 27 — Instrument parameters, gating parameters, and MFI histograms for FACS [file peerj-12-17397-s027.pdf]

Institution:
Protocol: siUSP22-3.PRO
Listmode Replay: Runtime Protocol
Analysis Date: 20-Feb-2024, 13:17:24
Settings File: hedaliushi230320.PRO, 27-Mar-2023, 16:41:26
Listmode File: siUSP22-3.LMD

Run Date: 27-Mar-23, 16:41:55
Sample ID: 00012031
User ID: liting
Acquisition Time/Events: 2.7s / 6000 (PROTOCOL)

Instrument SN: RAS11006 Software Version: CXP

(F1)[A] siUSP22-3.LMD : FS Lin/SS Lin - ADC

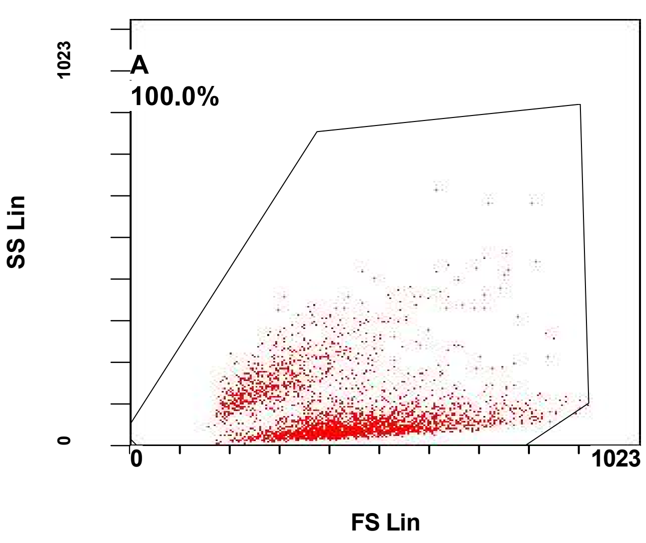

(F1)[A] siUSP22-3.LMD : FL1 Log/FL3 Log - ADC

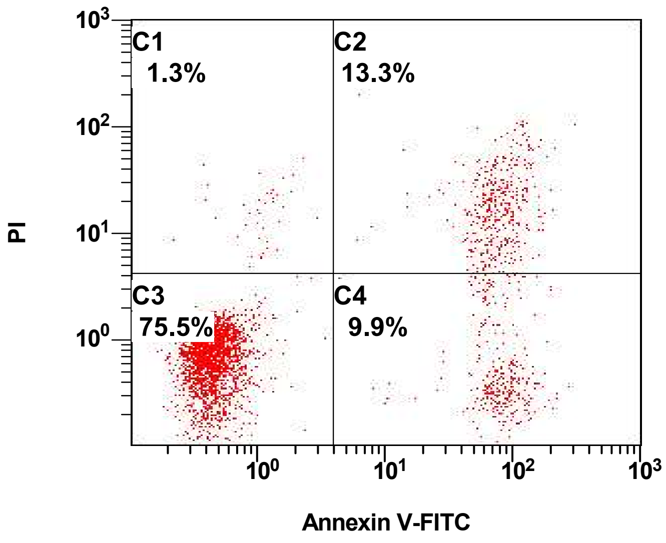

Statistical Analysis

PROGRAM INFORMATION

File:- siUSP22-3.LMD

Gate:- A [A]

Compensation:- Advanced

Filename:- siUSP22-3.LMD

Mean Calculation Method:- LOG-LOG

Region	Number	%Total	%Gated	X-Mean	Y-Mean
ALL	5935	98.92	100.00	19.5	4.23
ALL	5935	98.92	100.00	430	84.7
A	5935	98.92	100.00	430	84.7
C1	75	1.25	1.26	1.18	23.3
C2	788	13.13	13.28	80.5	24.5
C3	4483	74.72	75.53	0.471	0.802
C4	590	9.83	9.94	85.1	0.789
